# Novel use of a Weerda laryngoscope for transoral excision of a cervical ganglioneuroma: a case report

**DOI:** 10.1186/1752-1947-6-88

**Published:** 2012-03-26

**Authors:** Hidenori Yokoi, Atsushi Arakawa, Ayako Inoshita, Katsuhisa Ikeda

**Affiliations:** 1 Departments of Otolaryngology Head and Neck Surgery, Juntendo University School of Medicine, Tokyo, Japan; 2 Departments of Otolaryngology Head and Neck Surgery, Kyorin University School of Medicine; 3 Departments of Pathology, Juntendo University School of Medicine, Tokyo, Japan

## Abstract

**Introduction:**

A ganglioneuroma is a benign neoplasm arising from neural crest cells of the sympathetic nerve fibers and is most commonly seen in the posterior mediastinum or retroperitoneum. Although very uncommon, ganglioneuromas must be included in the differential diagnosis of neck masses. In young adult women, neck incisions made for excision of these benign tumors should be avoided whenever possible.

**Case presentation:**

We herein describe the case of a 19-year-old Japanese woman with a ganglioneuroma. The tumor was found in the parapharyngeal space, an unusual location. A fine-needle aspiration biopsy was performed but was considered inadequate to make a definitive diagnosis, so the asymptomatic lesion was surgically excised using a Weerda laryngoscope. The lesion measured 4 × 3 cm in size and was encapsulated. A pathological analysis showed the presence of two distinct cell types, ganglion cells and Schwann cells, embedded in a loose myxoid stroma. The final diagnosis was a ganglioneuroma.

**Conclusion:**

A complete excision was made possible by using a transoral approach with a novel use of the Weerda laryngoscope. Although its applicability to specific cases depends on the location, size and nature of the tumor, we believe that the Weerda laryngoscope will continue to be useful for performing transoral surgery for cervical tumors.

## Introduction

Ganglioneuroma is the most differentiated benign counterpart of neuroblastoma and originates similarly from neural crest cells that normally migrate into the adrenal medulla and sympathetic ganglia [1,2]. Ganglioneuroma can be found anywhere along the sympathetic chain, but is most commonly located in the posterior mediastinum and retroperitoneum [3,4]. Clinically, the signs and symptoms of cervical ganglioneuromas are usually related to the mass effect and nerve dysfunction, but these tumors often present as swelling with no specific symptomatology [2], as in the present case.

We herein describe a case of ganglioneuroma that was found in the parapharyngeal space, an unusual location [3,5]. Complete excision was made possible by using a transoral approach with a novel use of the Weerda laryngoscope. We also review the pertinent recent literature and discuss our findings.

## Case report

**Figure 1:**
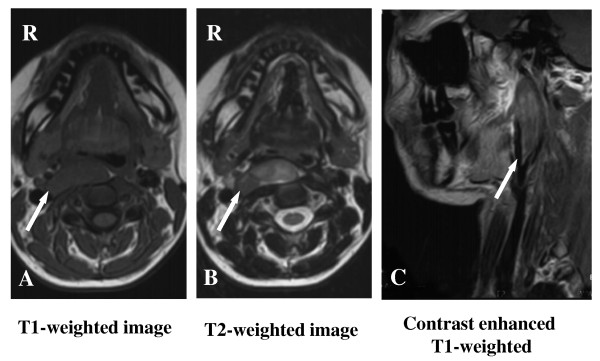
**MRI performed before surgical resection. (A) **MRI showing that the tumor has low-signal intensity on T1-weighted images. **(B) **Heterogeneous high-signal intensity on T2-weighted images. **(C) **The tumor exhibits an uneven contrast effect on T1-weighted coronal images. Arrows indicate a tumor.

**Figure 2:**
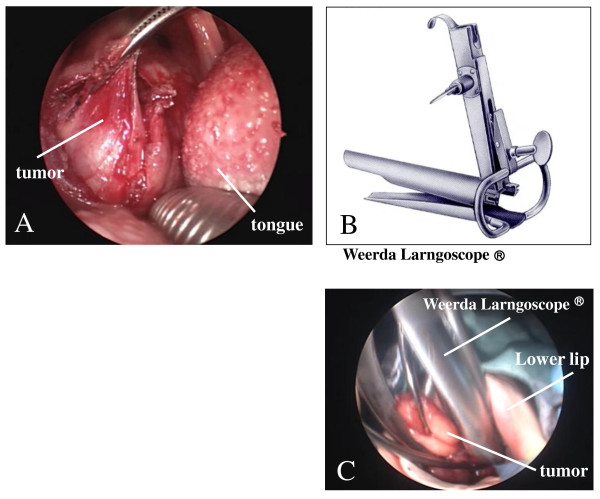
**Excision performed via the transoral approach with a novel use of the Weerda laryngoscope. (A) **Field of view of the oropharyngeal area obtained by using a Davis mouth gag. **(B) **Weerda laryngoscope. **(C) **Total excision made possible by use of the Weerda laryngoscope.

A 19-year-old Japanese woman presented to our hospital complaining of a mass in the middle of the pharynx, but without any systemic signs or symptoms. A mass measuring approximately 25 mm in diameter was noted in the middle of the pharynx. The mass was elastic in consistency, tender, and mobile, with a normal overlying oral mucosa. The patient's general condition and family history were unremarkable. A plain computed tomographic (CT) scan of the neck showed a low-density, homogeneous, neoplastic lesion occupying the right parapharyngeal space. A slight partial contrast effect was observed. Magnetic resonance imaging (MRI) showed the tumor to have low signal intensity on T1-weighted images and heterogeneously high signal intensity on T2-weighted images. The tumor exhibited an uneven contrast effect on the T1-weighted coronal images (Figure 1). A fine-needle aspiration biopsy was performed to rule out any malignancies but was inadequate to make a definitive diagnosis. For definitive diagnosis and treatment, a total resection of the tumor was performed while the patient was under general anesthesia. Complete excision using a transoral approach was possible by the use of a Weerda laryngoscope. Specifically, as in an excision of the palatine tonsils, we used a Davis mouth gag to obtain a clear field of view of the oropharyngeal area and then made a longitudinal incision in the center of the tumor to separate it from the mucosa (Figure 2A). However, the surgical field became difficult to visualize adequately while the deep portions and the outside of the tumor were separated, so the field of view was expanded by inserting a Weerda laryngoscope and slightly opening its tip, thereby making a total excision possible (Figures 2B and 2C).

**Figure 3:**
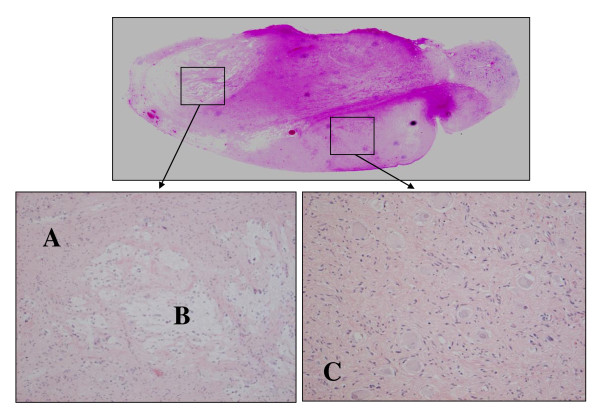
**Pathological findings. (A) **Part of the collection of maturing ganglion cells. **(B) **Part of the Schwann cell and collagen fiber. **(C) **Image showing part of the mixture of ganglion cells, the Schwannoma cell and collagen fiber. The histological findings were consistent with ganglioneuroma, maturing subtype, as a Schwannian stroma-dominant neuroblastic tumor.

A gross pathological examination revealed a well-circumscribed 72 × 33 × 11-mm tumor with a fibrous capsule. The cut surface was whitish and exhibited a whorled pattern. A histopathological examination showed the tumor to be composed predominantly of nodular neurophilic ganglioneuromatous stroma with a minor component that consisted of collections of maturing ganglion cells which were unevenly distributed. The fibrous capsule of the tumor was thinned in areas, but no extracapsular extension was identified. The histological findings were consistent with ganglioneuroma, maturing subtype (Schwannian stroma-dominant neuroblastic tumor) (Figure 3).

After surgery, the patient exhibited left palpebral ptosis and anisocoria with ipsilateral mydriasis typical of Horner's syndrome. These symptoms resolved completely within three months.

Ultrasonography of the abdomen was performed to exclude any visceral involvement. The patient's postoperative course was satisfactory, and she was discharged with no difficulties. No local recurrence or distant metastases have been observed during the five years since her surgery.

## Discussion

Sympathetic nervous system tumors include neuroblastomas, ganglioneuroblastomas and ganglioneuromas [5]. All are neural crest cell derivatives and are considered to be different maturational steps of a unique neoplasm [5]. The clinical behavior of these tumors reflects their state of differentiation. Neuroblastoma and ganglioneuroblastoma are malignant neoplasms [6], and the histologic grade of and prognosis for a given tumor are determined by the proportion of neuroblastoma elements [5,7] (Table 1). A distinctive feature of a maturing subtype ganglioneuroma is that the neuroblastomatous foci do not form any distinct microscopic nests as they do in a ganglioneuroblastoma. Instead, individual neuroblastic cells merge with the predominantly ganglioneuromatous stroma [7]. This subtype, which was observed in our patient, has previously been described as "stroma-rich, well-differentiated" according to the original Shimada classification [7].

**Table 1:** Prognostic evaluation of neuroblastic tumors^a^

International Neuroblastoma Pathology Classification	Tumor description	Original Shimada classification	Prognostic group
Neuroblastoma	Schwannian stroma-poor	Stroma-poor	
Favorable		Favorable	Favorable
< 0.5 years	Poorly differentiating or differentiating and low or intermediate MKI tumor		
1.5 to 5 years	Differentiating and low MKI tumor		
Unfavorable		Unfavorable	Unfavorable
< 1.5 years	Undifferentiated tumor		
	High MKI tumor		
1.5 to 5 years	Undifferentiated or poorly differentiated tumor		
	Intermediate or high MKI tumor		
≥ 5 years	All tumors		
Ganglioneuroblastoma, intermixed	Schwannian stroma-rich	Stroma-rich intermixed (favorable)	Favorable
Ganglioneuroma	Schwannian stroma-dominant		Favorable
Maturing		Well-differentiated (favorable)	
Mature		Ganglioneuroma	
Ganglioneuroblastoma, nodular	Composite Schwannian stroma-rich/stroma-dominant and stroma-poor	Stroma-rich nodular (unfavorable)	Unfavorable

^a^MKI: mitosis-karyorrhexis index.

Ganglioneuromas are composed only of mature elements and rarely if ever metastasize, so the prognosis is usually favorable [2,7].

Ganglioneuromas are usually found in the posterior mediastinum or in the retroperitoneum [3,4]. A report in which tumors were classified according to their anatomical distributions showed that only one of 88 patients had a tumor in the parapharyngeal region. The most common sites were the mediastinum (34 cases) and the retroperitoneum (27 cases) [3].

Although very uncommon, ganglioneuromas must be included in the differential diagnosis of neck masses, along with infectious cervical adenitis, branchial cleft cyst and some other malignancies, such as other variants of neuroblastic tumors, sarcoma and malignant lymphoma [2]. Surgical excision is the treatment of choice in these cases, both to confirm the diagnosis and to prevent any further tumor growth and consequent compression of the adjacent structures.

Numerous approaches for resection of parapharyngeal space neoplasms have been described, including the transcervical approach, the transparotid approach, the transcervical-transpharyngeal approach, the infratemporal fossa approach and combinations of the these [8,9]. The location, size and pathological type determine the choice of surgical approach [10]. Transoral resection of a superomedial parapharyngeal benign neoplasm, with decreased morbidity compared with that of traditional approaches, has also been reported [11]. In contrast to external approaches, the transoral approach does not require dissection in proximity to facial nerve branches; furthermore, the potential for postoperative salivary fistula present with transparotid techniques is avoided. In addition to these advantages, neck wounds can be avoided and patients can resume oral intake after surgery, making short hospitalizations possible [11]. However, before surgery, it is important to communicate with the patient about the relative advantages and disadvantages of external and transoral approaches, as the latter allow less exposure and carry risks of hemorrhage, damage to cranial nerves (including Horner's syndrome and first-bite syndrome) and tumor spillage.

In our present case of a 19-year-old woman, a tumor was observed in the neck and was found to be prominent on the right side of the oropharynx when her mouth was open. To avoid performing an excision through an incision if possible, we attempted an excision using a transoral approach with a Weerda laryngoscope.

The Weerda laryngoscope has recently been reported to be useful for the removal of early esophageal lesions [12] and embedded esophageal foreign bodies [13]. In this study, we report the first case of a patient from whom a tumor of the parapharyngeal space was excised using a transoral approach with a Weerda laryngoscope.

## Conclusion

Although very uncommon, ganglioneuromas must be included in the differential diagnosis of neck masses. Although its applicability to individual cases may depend on the location, size and nature of the tumor, we believe that the Weerda laryngoscope will continue to be useful for transoral surgery for cervical tumors.

## Consent

Written informed consent was obtained from the patient for publication of this case report and any accompanying images. A copy of the written consent is available for review by the Editor-in-Chief of this journal.

## Competing interests

The authors declare that they have no competing interests.

## Authors' contributions

HY, AI and KI performed surgery on the patient. AA analyzed the pathological findings as a pathologist. HY drafted the manuscript. All authors read and approved the final manuscript.
